# Cryopreservation of tendon tissue using dimethyl sulfoxide combines conserved cell vitality with maintained biomechanical features

**DOI:** 10.1371/journal.pone.0215595

**Published:** 2019-04-19

**Authors:** Eva Hochstrat, Marcus Müller, Andre Frank, Philipp Michel, Uwe Hansen, Michael J. Raschke, Daniel Kronenberg, Richard Stange

**Affiliations:** 1 Department of Regenerative Musculoskeletal Medicine, Institute for Musculoskeletal Medicine, University Hospital Münster, Westfälische Wilhelms-University, Münster, Germany; 2 Department of Trauma-, Hand-, and Reconstructive Surgery, University Hospital Münster, Münster, Germany; 3 Department of Molecular Medicine, Institute for Musculoskeletal Medicine, Westfälische Wilhelms-University, Münster, Germany; Mayo Clinic Minnesota, UNITED STATES

## Abstract

Biomechanical research on tendon tissue evaluating new treatment strategies to frequently occurring clinical problems regarding tendon degeneration or trauma is of expanding scientific interest. In this context, storing tendon tissue deep-frozen is common practice to collect tissue and analyze it under equal conditions. The commonly used freezing medium, phosphate buffered saline, is known to damage cells and extracellular matrix in frozen state. Dimethyl sulfoxide, however, which is used for deep-frozen storage of cells in cell culture preserves cell vitality and reduces damage to the extracellular matrix during freezing. In our study, Achilles tendons of 26 male C57/Bl6 mice were randomized in five groups. Tendons were deep frozen in dimethyl sulfoxide or saline undergoing one or four freeze-thaw-cycles and compared to an unfrozen control group analyzing biomechanical properties, cell viability and collagenous structure. In electron microscopy, collagen fibrils of tendons frozen in saline appeared more irregular in shape, while dimethyl sulfoxide preserved the collagenous structure during freezing. In addition, treatment with dimethyl sulfoxide preserved cell viability visualized with an MTT-Assay, while tendons frozen in saline showed no remaining metabolic activity, indicating total destruction of cells during freezing. The biomechanical results revealed no differences between tendons frozen once in saline or dimethyl sulfoxide. However, tendons frozen four times in saline showed a significantly higher Young’s modulus over all strain rates compared to unfrozen tendons. In conclusion, dimethyl sulfoxide preserves the vitality of tendon resident cells and protects the collagenous superstructure during the freezing process resulting in maintained biomechanical properties of the tendon.

## Introduction

Biomechanical and biomolecular research on tendon tissue is of increasing scientific interest and relevance. As part of the musculoskeletal system, tendon tissue is able to transmit energy produced by muscles to the bones due to its typical composition of fibrillar collagens and other proteins, and hence, enables joint and limb movement[1]. It also buffers impact load to protect the muscle from potential damage by storing energy and subsequently releasing it gradually[2]. Tendon tissue can serve these functions due to its typical structure including strict hierarchical collagenous composition as well as low cellularity and vascularization resulting in low metabolic activity[3]. The latter often leads to a protracted healing process and in some cases incomplete recovery following ruptures, cutting damages or tendinopathies and thus a decline of biomechanical properties[4] resulting in re-ruptures, chronic pain and restricted mobility[5,6]. Research focusing on tendon tissue therefore investigates new treatment strategies such as improved suture techniques, tendon engineering and the use of auto-, allo- and xenografts[7]. Improving and evaluating these treatment strategies is commonly executed with animal models, providing the possibility to include gene alterations or pharmacological treatments and test them under standardized conditions[8]. Analysis of animal tendons often includes biomechanical testing, to evaluate functional outcome of the treatment. Therefore, it is common practice to store tendon tissue, mostly deep-frozen, until use to ensure that all harvested tendons are analyzed under equal conditions.

Cryopreservation in scientific as well as in clinical use should alter the frozen tissue as little as possible. Unfortunately, freezing temperature[9] and duration of the deep-frozen state[10], as well as water content and the medium used and therefore especially multiple freeze-thaw-cycles, can alter the structural properties and lead to varying biomechanical outcomes[11].

For cryoconservation tendons are commonly wrapped with gauze soaked in phosphate buffered saline (PBS) without adding any cryoprotectants[12]. This isotonic buffer solution ensures cell vitality during experimental use in a fluid state. Freezing, however, leads to the formation of ice-lenses, damaging the ECM and destroying the tenocytes, resulting in modified biomechanical properties compared to fresh tendons[13].

Dimethyl sulfoxide (DMSO) is a compound used in cell culture for cryopreservation, since it has multiple cryoprotective effects. In liquid state DMSO functions as a buffer, similar to PBS. The effect that distinguishes DMSO from PBS is revealed when it comes to freezing of the solution.

DMSO reduces the freezing point of aqueous solutions. Additionally, interactions with the cell membrane have been described leading to a preserved integrity of the cell membrane during freezing and thawing. Additionally, DMSO increases the viscosity of the cytosol which results in the formation of none or at least smaller ice lenses and the reduction of dehydration of the cells during freezing which all in all reduces the damage put on cell which enables cell survival and protects the extracellular matrix[14]. Egli et al. [12] showed that cells in femora of neonatal mice remained vital, when frozen in presence of DMSO. Similar positive effects on the vitality of tenocytes could be shown using 10% DMSO in Dulbecco's Modified Eagle's Medium (DMEM) [15]. Preserving cell vitality and integrity in the tendon during cryoconservation will contribute to the biomechanical capacity of the tendon and might additionally conserve tendon cells for further investigations. To the best of our knowledge, research on the influence of DMSO on biomechanical properties of tendons has not been conducted before.

Therefore, this study investigates the potential positive effect of DMSO on the ECM and cells in murine Achilles tendons resulting in enhanced biomechanical features compared to tendons stored in PBS. Furthermore, matrix-protecting potential of DMSO in a scenario of more than three freeze-thaw-cycles was taken into consideration, since it has already been shown that three freezing cycles lead to significant reduction of maximum load in rabbit Achilles tendons. An additional application of up to ten freezing-cycles on the contrary does not lead to further impairment of the biomechanical features additionally[13]. Consequently, an application of more than two freeze-thaw cycle would have been suitable to cause intensified damage to the tendon tissue. A count of four freezing cycles was therefore chosen to securely damage the tendon consistently.

## Material and methods

### Animal handling and sample preparation

C57/Bl6J mice were obtained from Jackson laboraties (over Charles River, Sulzfeld Germany) and housed in the central animal facility of the university hospital Münster (ZTE). Twenty-six male mice (aged 20 weeks) were directly euthanized by cervical dislocation immediately after narcosis with 5% isoflurane in accordance with the National Institute of Health guidelines for the use of laboratory animals. The intuitional permission to sacrifice animals for scientific purposes was granted to our institute by the LANUV (North-Rhine Westphalia) under the reference 84–02.05.50.15.005 which is sufficient under German animal protection laws.

The hind limbs were shaved to reduce interference of the fur with the microscopic imaging during biomechanical testing and subsequently removed. Both Achilles tendons were carefully dissected under microscopic view. The insertion on the calcaneus was left intact. Both Achilles tendons were used. Tendons were randomized in 5 groups via an online randomizing tool (random.org, Dublin, Ireland) ensuring that tendons of one animal did not appear in the same group. Groups included at least seven tendons per group. Data Sets in which premature rupture, slippage from the clamps or other deviations from correct course of protocol happened were excluded. Group 1 (Control) was tested directly after preparation. Group 2 was stored without cryoprotection in PBS (Dulbecco’s PBS, Sigma Aldrich, Taufkirchen, Germany). Group 3 was frozen in DMSO (20% in PBS, Sigma Aldrich, Taufkirchen, Germany). Groups 4 and 5 underwent four freeze-thaw-cycles, in which group 4 was kept in PBS and group 5 in DMSO. We have chosen 20% DMSO since it is commonly used in cell culture to preserve cells for nitrogen culture. Tendons, undergoing freezing were wrapped in PBS or DMSO soaked gauze and frozen at about -20°C. One freezing cycle included a freezing time of 2 hours. Thawing was performed at room temperature for 1 hours. Specimens were kept frozen until usage for varying periods up to two weeks mimicking actual procedure during biomechanical testing of animal tendons.

### In-situ cell survival

Additionally, three murine Achilles tendons were carefully dissected. Tendons were assigned to three groups, representing unfrozen tendons and tendons frozen once in PBS or DMSO. Tendons were incubated in cell-culture medium (αMEM, Biozol) with 0.5 mg/ml 3-(4,5-dimethylthiazol-2-yl)-2,5-diphenyltetrazolium bromide (MTT) for 3 hours. This agent is reduced by metabolic active cells to its formazan form, which is violet in coloration. The extent of violet staining was used as an indicator for cellular survival. The intensity and distribution of the staining was compared.

### Isolation of tenocytes

Tendons were frozen as previously described. Afterwards the Achilles tendon (N = 3) were pre-incubated for 10 minutes in Dulbecco’s modified eagle medium (DMEM) with 0.1 mg/ml collagenase I (Sigma Aldrich, Taufkirchen, Germany) to remove the tendon sheath fibroblasts. The tendons were put in culture using DMEM with 10% fetal bovine serum, 100 u/ml penicillin/streptamicin, 2 mM glutamine and 1 mM L-Ascorbat-2-phosphat. After 7 days, cells were photographed using an Olympus CKX53 microscope. After 8 days, an MTT assay with the resident cells for 3h was performed as described above. The blue colorant was solved using 10 ml of SDS solution (20%) mixed with 10 ml dimethyl formamide and its absorbance was quantified at 570 nm with 630 nm as reference wave length.

### Quantitative real-time PCR

Tendons have been dissected and frozen as previously described. 10 μl collagenase solution (0.1 mg/ ml in PBS) per mg tendon was added and the tissue was digested for 30 min at 37°C. The same quantity RJL buffer (Qiagen, Hilden Germany) was added. The RNA was purified using the Qiagen Micro RNA purification kit according to manufacturer’s protocol. The RNA material of 20 mg tendon was purified and used further on. The primer sets (Metabion Planegg/Steinkirchen Germany), used for quantitative real time PCR (qRT-PCR), are acacccagcccaaacagat for scleraxis forward and tctgtcacggtctttgctca for scleraxis reverse. qRT-PCR was performed using the DyNAmo Flash SYBR Green qPCR Kit (Biozym, HessischOldendorf Germany). Scleraxis is a gene marker produced in tenocytes at high levels [16]. Each experiment included a negative control using water as template. The data were normalized by using the same sample mass and by setting the CT of the fresh tendon to 100%.

### Electron microscopy

Tendons were fixed in 2% formaldehyde (v/v) and 2.5% glutaraldehyde (v/v) in 100 mM cacodylate buffer, pH 7.4, at 4°C overnight. After washing with PBS, samples were postfixed in 0.5% osmium tetroxide with 1% potassium hexacyanoferrate (III)) in 0.1 M cacodylate buffer for 2 hours. After washing the samples with water, they were dehydrated in a descending ethanol series. Samples were then incubated in propylene oxide twice and embedded in Epon. Ultrathin longitudinal sections were collected on grids and contrasted with 2% uranyl acetate. Electron micrographs were taken with a Philips EM-410 electron microscope at 60 kV using imaging plates (Ditabits, Pforzheim, Germany). The electron micrographs were evaluated quantitatively as comparison of frozen tissue to fresh unfrozen tissue according to Chen et al.[13].

### Biomechanical set-up

The tendons were thawed at room temperature. The tendon tissue reached room temperature 30 minutes after being removed from the freezer, which was confirmed using an infrared thermometer (VoltcraftIR 1000-CAM, Conrad, Hong Kong, China). Afterwards, tendons were mounted in a custom made test-set-up similar to O’Brien et al.[17]. Displacement-controlled elongation was performed with the axial test-bench LM1 (TA Instruments/ElectroForce, New Castle, USA).

The calcaneus was clamped under a metal pin. The proximal site of the tendon was fixed in a plastic clamp under use of sandpaper. Specimens were transferred to a PBS-bath at room temperature. Tendon dimensions including length and width were measured with cameras from two angles. Three cross-sectional areas of the mid-region were calculated and averaged. The testing protocol was similar to Dourte et al.[18], which has been used before to analyze dynamic biomechanical features of tendon tissue. It contains a preconditioning-phase with a cyclic loading between 0.5% and 1.5% strain at 0.25Hz. Samples were stretched to 4%. A 10-minute Stress-relaxation-test followed by subsequent cyclic loading in a frequency sweep (0.01 Hz, 0.1 Hz, 1Hz, 5 Hz) with an amplitude of 0.125% strain was performed. Analog tests were conducted at strain levels of 6% and 8%. Finally, tendons were stressed until failure in a displacement-controlled load-to-failure-ramp with a speed of 0.1% strain per second.

After measurement, values for dynamic Young’s modulus were mathematically standardized to an individual preload of 0.5MPa.

Dynamic Young’s Modulus was calculated from the amplitude ratio of the stress-time-curve/strain-time-curve. The dynamic Young's modulus is known to represent the tissues resistance against deflection. Static Young's Modulus was calculated from the linear-elastic region of the load-to-failure-curve.

Statistical analysis was performed via two-way ANOVA (GraphPad Prism 7, GraphPad Software, San Diego, USA) for analysis of dynamic and static Young's modulus. Linear regression was performed to check for general influence of freezing medium and number of freeze-thaw-cycles. A p-value of >0.05 was considered statistically significant.

## Results

### In situ cell survival

**Fig 1:**
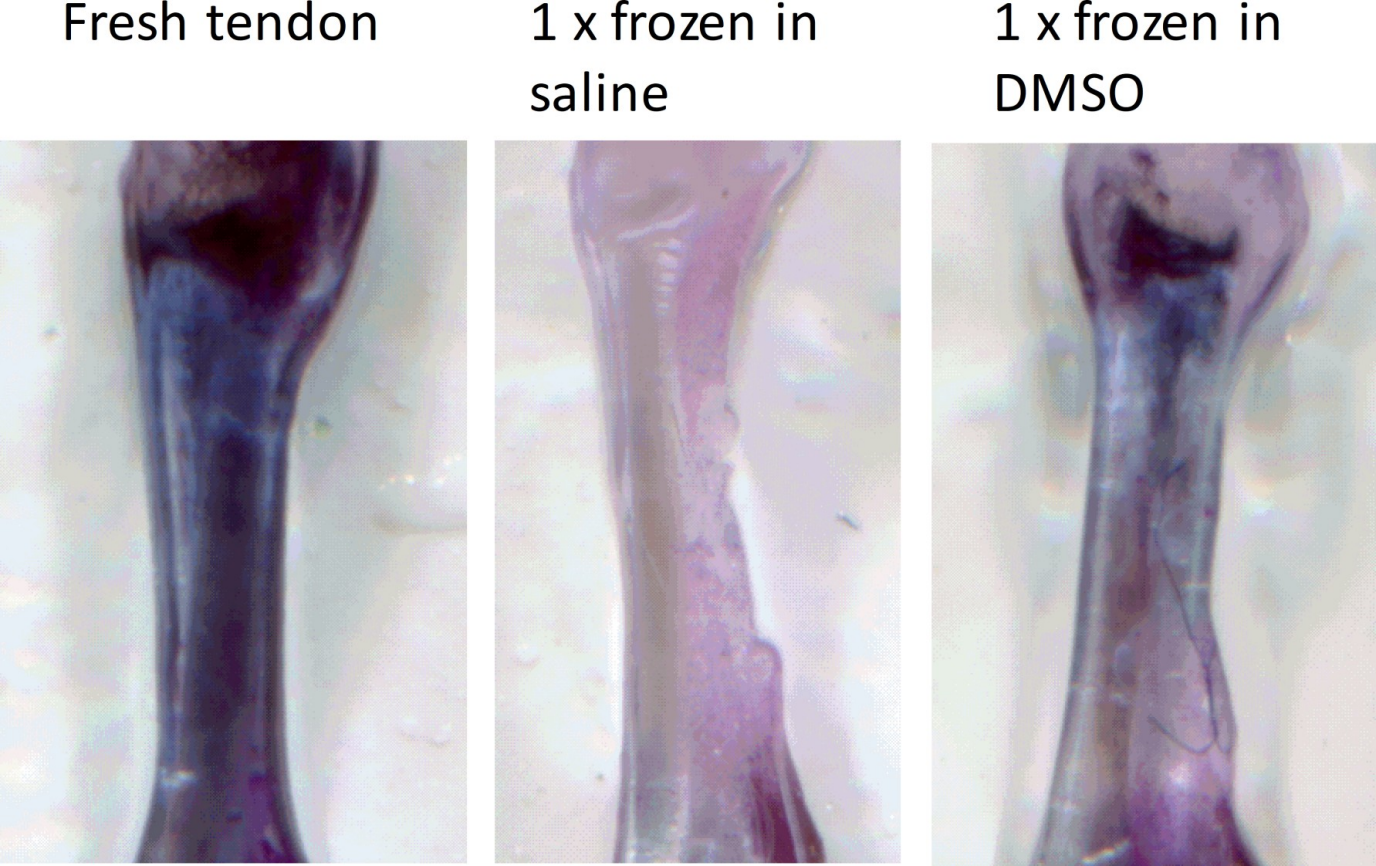
Freezing tendons in DMSO retains metabolic active cells in the tissue. Dissected tendons were frozen either in PBS or in 20% DMSO containing PBS at -20°C and thawed at room temperature. The cells were incubated in αMEM for 3h containing 10% MTT reagent. Violet coloration shows metabolic active cells.

For assessing cell survival, comparison of the blue staining after incubation with MTT was performed (Fig 1). Fresh tendons which did not undergo freezing were stained blue across the whole tendon substance and served as control group. Tendons frozen in DMSO showed a less distinct but explicit staining, mostly in the transition zone between tendon and calcaneus. The staining of tendons frozen in PBS could be seen at best in the surrounding tissue of the tendon. This points to a complete destruction of the tenocytes. In contrast using DMSO as a freezing medium sustains the vitality of a great quantity of tendon resident cells.

**Fig 2:**
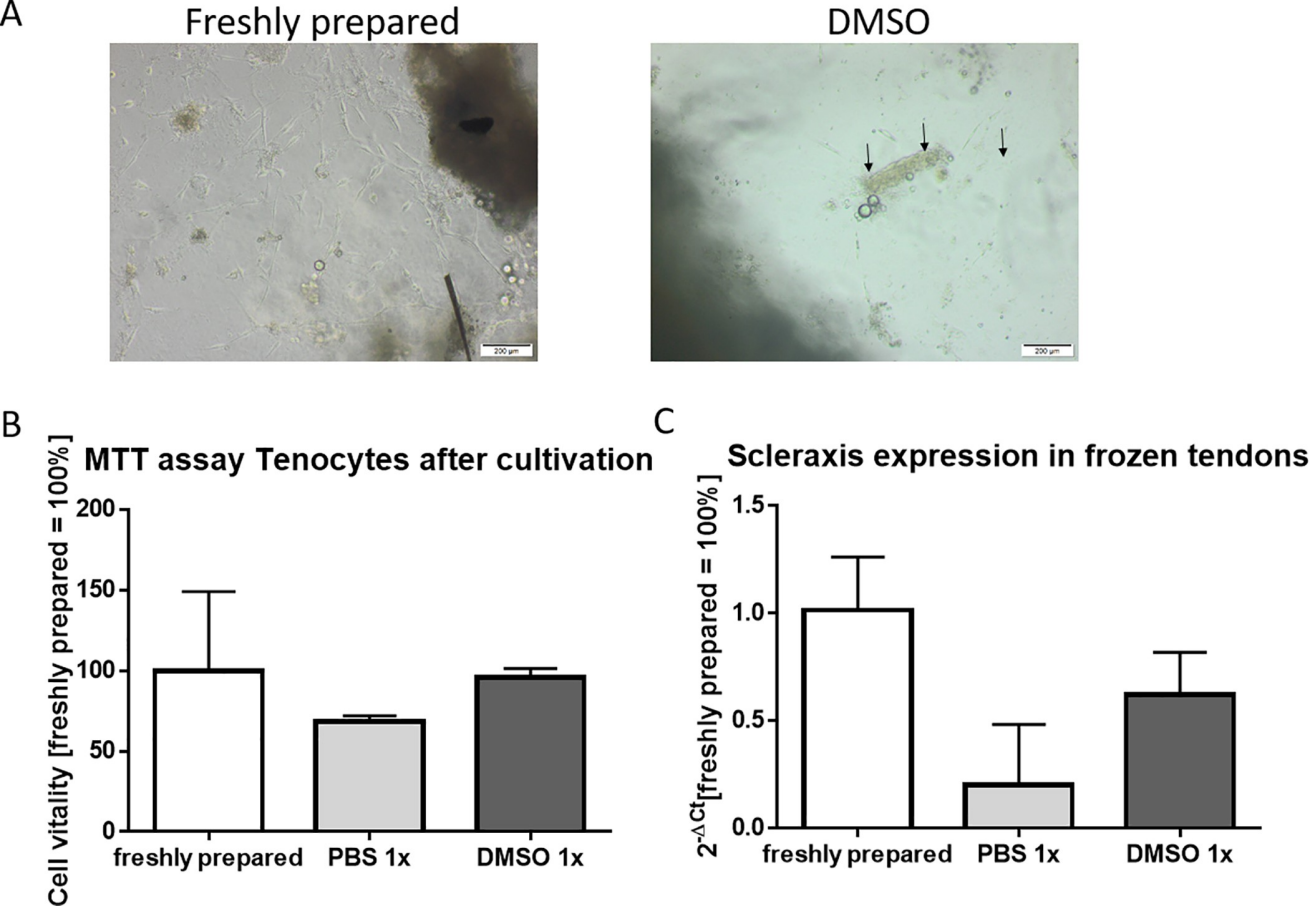
Freezing tendons in DMSO retain scleraxis positive cells, which can be put in cell culture. After 7 days of cultivation, tendon-derived cells can be detected either in freshly cultivated as well as in in DMSO frozen tendons (A). Quantification of MTT -assay of tendon derived cells after 8 days of cultivation (B). Quantitative real-time PCR of the resident cells from freshly prepared, in PBS and DMSO-frozen tendons, testing the scleraxis expression(C).

Additionally, tendons were cultivated in cell culture for 7 days and cell growth was evaluated visually and quantitatively by MTT-Assay and qPCR of Scleraxis gene expression. Fresh tendons served as a reference. From tendons frozen in PBS no cells were found migrating from the tendon tissue into the cultivation medium and attached to the base of the dish. Consequently, after 7 days of cultivation, no cells were visible the quantification of the MTT-Assay were lower than the measured values for fresh tendons or those frozen in DMSO. Tendons frozen in DMSO showed less but although visible cell growth compared to fresh tendons (Fig 2A). In addition, measured vitality of the cells using the MTT-Assay showed values as nearly as high as the values assessed for fresh tendons (Fig 2B).

Quantification of the scleraxis gene expression showed for in PBS frozen tendon a neglectable amount of gene activity. On the other hand, the tendon frozen in DMSO had similar expression levels as the freshly prepared control pointing to the survival of a tenocyte population (Fig 2C).

### Electron microscopy

**Fig 3:**
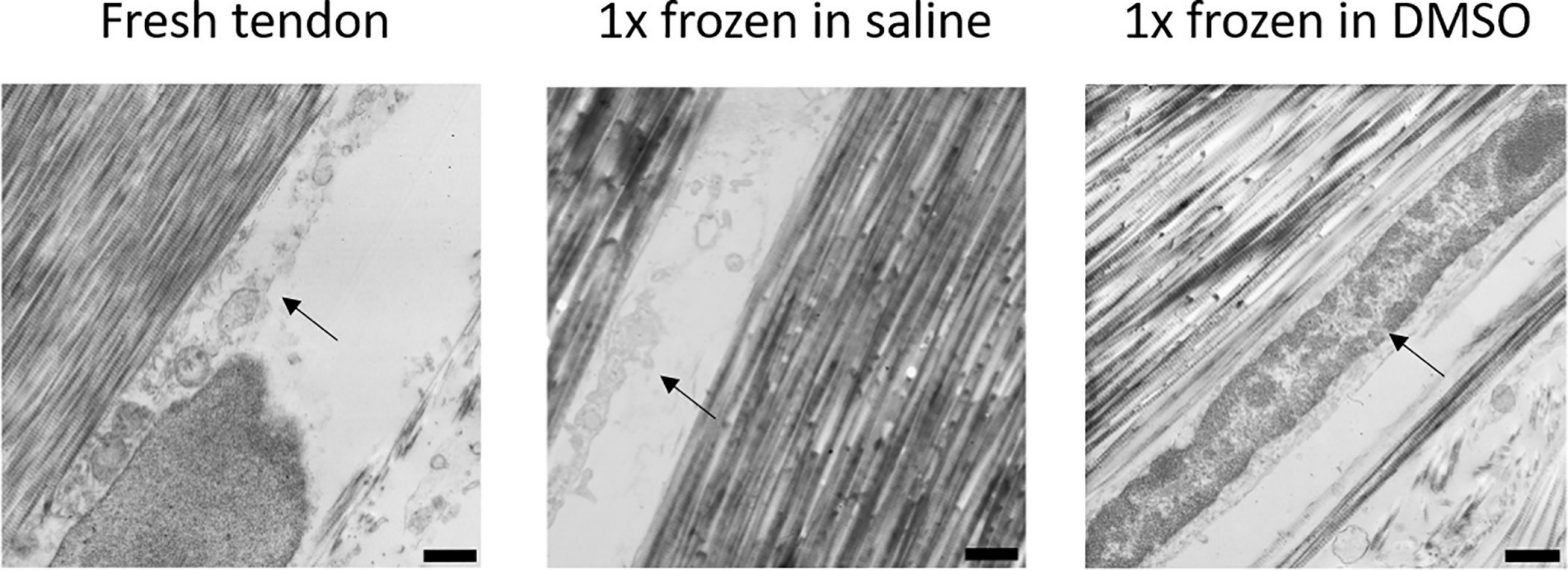
Freezing tendons in DMSO retains cells in-situ, which were destroyed when freezing in PBS. Transmission electron microscopy of Achilles tendon after freezing once using PBS or 20% DMSO in PBS as medium. Arrows mark matrix cavities housing tenocytes with either intact integrity or remaining debris.

An overview showed that the fresh tendon is containing tenocytes, which are embedded in the collagen matrix. These cells retained their integrity and organelles are visible. In tendons frozen in PBS we just found remnants of cells. In contrast, in tendons, which were frozen in DMSO we could find cells with properly formed cell shape and visible organelles comparable to the situation described in fresh tendons. This would indicate that the DMSO frozen sample contained viable cells. (Fig 3)

**Fig 4:**
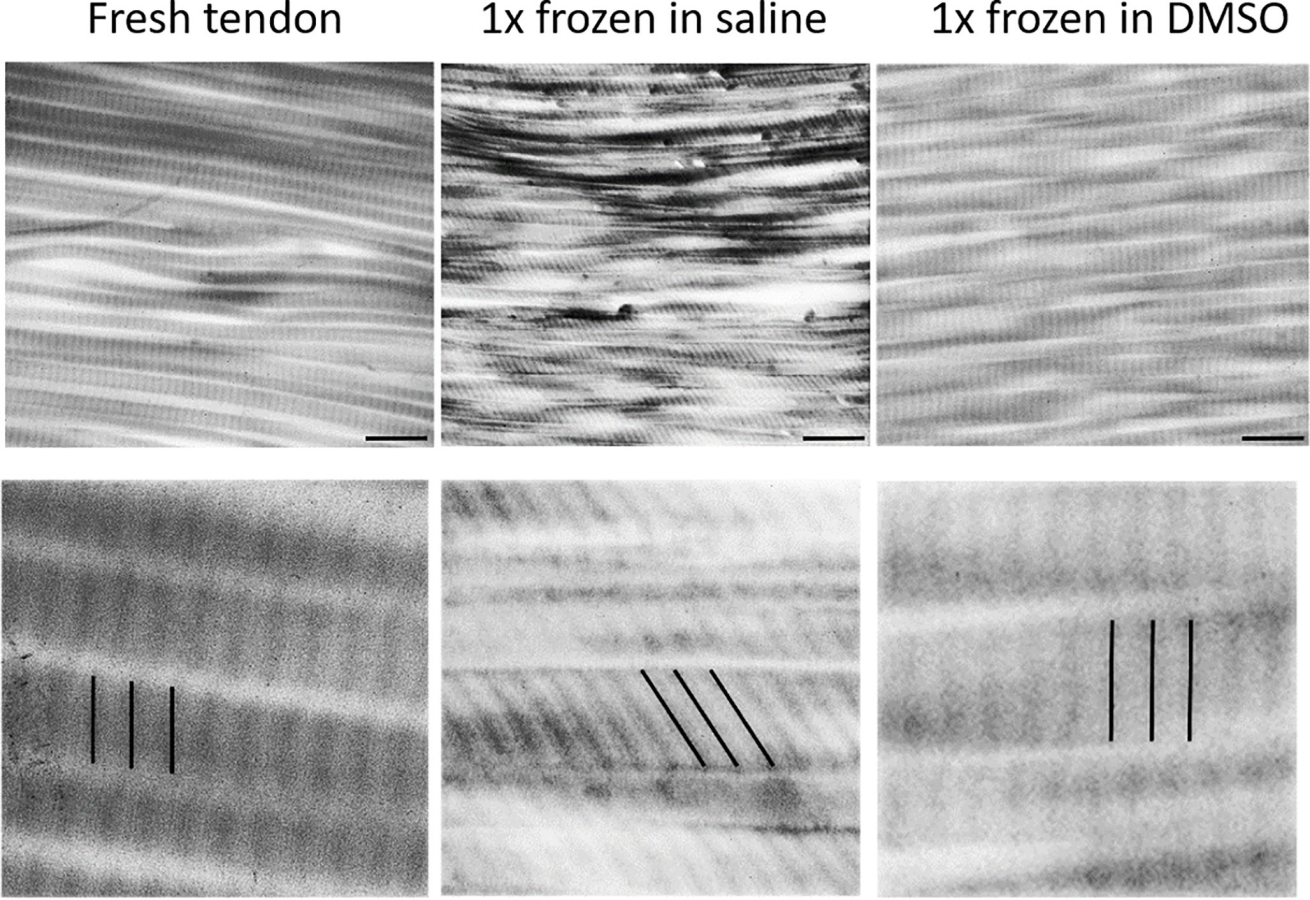
Collagen morphology of Achilles tendon is disturbed after freezing in PBS but not in DMSO. Transmission electron microscopy of Achilles tendon after freezing once using PBS or 20% DMSO in PBS as medium. Lines mark the orientation of bands caused by the D-period in relation to the fiber orientation in the detail images. Scale bar: 500 nm.

A comparison of the fibrillary structure of collagen in the tendon, visible in the electron micrographs, was made (Fig 4). Tendons, which did not undergo freezing served again as a control group and showed a regular smooth fibrillary collagen structure.

Similar appearance with a regular pattern could be seen in tendons frozen in DMSO.

In contrast, tendon tissue frozen in PBS showed a rough collagen structure with destruction of the surface compared to an unfrozen tendon. The typical D-periodicity of collagen fibrils- the band pattern occurring with an interval of 67nm- showed no orthogonal, but a diagonal main direction of the fibrils. This, in addition to the previous described alterations, provides evidences for a shift of collagen molecules within the collagen fibril, and therefore, damage at the molecular level caused by freezing.

### Biomechanical analysis

For evaluation, results were referenced to group 1 (freshly tested) for all strain rates (4%, 6% and 8%). In all groups a linear increase in dynamic Young's modulus at increasing strain rates was observed. Furthermore, it was noticeable that frequency affects the dynamic Young’s modulus, since increased frequency led to an increased Young's modulus within one strain level. Contrary to our expectations, there was no significant difference between tendons frozen in PBS compared to the control group for all strain rates regarding static and dynamic Young's modulus. In addition, no difference could be seen between tendons frozen in DMSO and the control group considering all measured biomechanical features.

Analysis of static Young's modulus revealed no differences between freshly tested tendons and tendons frozen once in PBS or DMSO. At a strain rate of 6% and a frequency of 1Hz dynamic Young’s modulus amounts 387±169,35 for group 1, 368,9±98,98 for group 2 and 386±131,71 for group 3. Group 4 showed a considerably higher static Young's Modulus (495±183,67 at 6% strain and 1Hz), which was not significant compared to group 1, whereas group 5 showed no significant differences to the freshly tested tendons (467,6±245,3 at 6% strain and 1Hz). At the frequency of 0.1 Hz Young’s modulus was also significant for comparison of group 1 and 4. At very low frequencies (0.01Hz) and high frequencies (5Hz) no significant difference could be detected.

**Fig 5:**
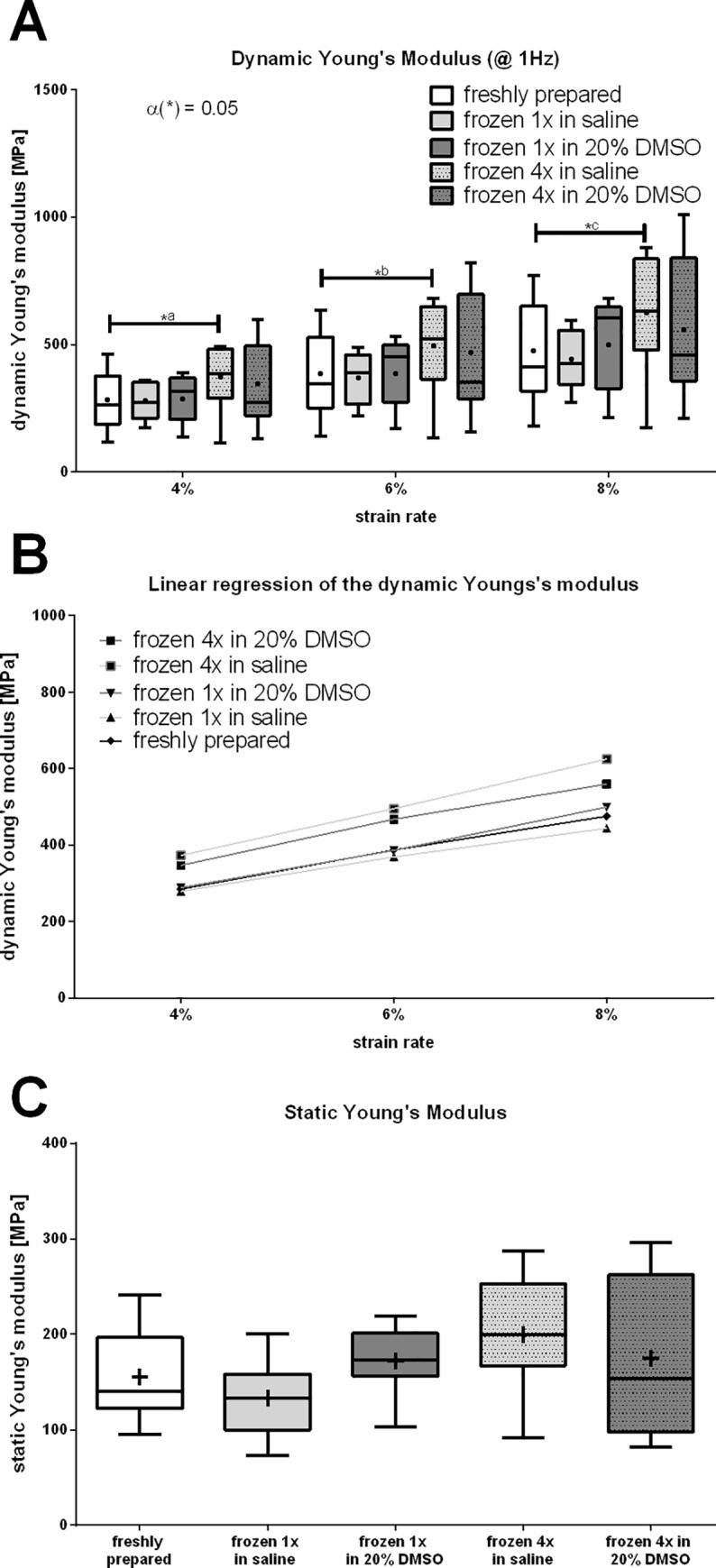
Dynamic biomechanical testing showed significant differences between tendons frozen in PBS four times and freshly tested tendons for the dynamic Young's modulus. Biomechanical testing was performed using a custom-made axial testing set-up (LM1, ElectroForce/TA Instruments). Dynamic Young's modulus calculated from the amplitudes of the dynamic testing at a frequency of 1Hz for all strain rates (A) is shown for freshly tested tendons, tendons frozen once in PBS or 20% DMSO in PBS as well as tendons frozen four times in PBS or 20% DMSO in PBS. A linear increase at increasing strain levels (B) was observed. The static Young's modulus was calculated from the linear region of the ramp-to-failure-curve (C).

Nevertheless, Group 4 and 5, which were frozen four times showed a wider deviation of measured values for dynamic Young’s modulus. Representative figures are shown for the frequency of 1Hz (Fig 5). Different frequencies showed similar results and are listed in the supplemental data (S1 Fig).

Noticeably, fresh tendons and tendons frozen once show nearly similar dynamic Young's modulus in all tested strain rates. At strain rates of 8% the measured values show a slightly higher deviation.

Tendons frozen four times in PBS showed significantly higher Young’s Modulus for all strain rates compared to the control group, implicating that tendons frozen four times in PBS show a higher resistance against deformation, resulting in a reduction of the tendons capability of buffering energy and its spring-like function at low strain rates. Tendons frozen in DMSO showed no significant differences. In general, Young's modulus assessed from tendons, which underwent four freeze-thaw-cycles showed a wider deviation and more inconsistent values.

Linear regression showed no significant influence of the freezing medium on Young's modulus (p = 0.748). Noticeably, the number of freeze-thaw-cycles showed significant influence (p <0.05).

## Discussion

In this study, we compared different protocols for cryoconservation of murine tendons for further evaluation. We demonstrated that freezing of tendon tissue in PBS already causes an impairment of the tendons resident collagen fibrils on a molecular level as well as a nearly complete void of cells. The use of DMSO, on the contrary, led to maintained vitality and collagenous structure within the tendon tissue.

It has already been shown that DMSO preserves porcine tenocyte vitality during cryoconservation[15]. In this study we established a novel simple method to evaluate tenocyte viability incubating whole tendons in MTT and evaluating the staining of the tissue. Hence, this method is suitable for easy assessment of viability of tendon residential cells. We verified the finding we postulated by using the MTT-assay of the whole tendons tissue, namely that there are no vital cells in tendons frozen in PBS, by putting fresh tendons and tendons frozen in PBS and DMSO in cell culture. We subsequently evaluated cell survival visually and by quantifying an MTT-assay of the cultivated cells and qPCR of Scleraxis gene. All these different methods showed, that in contrast to tendons frozen in DMSO, in tendons frozen in PBS no cell growth was apparent. Measured values in the quantification of the MTT-assay might regarding the absence of cells in the culture flask and in summation of the results be due to background coloring.

The evaluation of the electron micrographs revealed no differences between fresh tendons and tendons frozen in DMSO. Tendons that were stored in PBS showed striking destruction of the collagenous structure. Here, a shift in the orientation of collagen molecules became visible since the D-period of the collagen fibers seems to be altered. An oblique pattern instead of a strictly orthogonal orientation of the pattern appeared. Several explanations are possible including destruction of the collagenous structure due to formation of ice lenses or disruption of the structure by destruction of the residential cells in the tendon. Ding et al.[19] were able to show that multiple freeze-thaw-cycles applied to collagen solution led to formation of a microporous structure in which the pores were mainly filled with water during the frozen storage. The porous network appeared to become smaller and more regular applying more freezing cycles, which could be due to increasing number of entanglement points during the freezing process. In our study, whole tendon tissue was analyzed, which consists mainly of collagen type I. Regarding the previously mentioned findings it seems obvious that freezing has to have an effect on the collagenous structure, which seems to be reduced by the use of DMSO resulting in preserved structure of collagen.

For evaluating the collagenous structure, longitudinal sections were analyzed, since transverse sections revealed no differences. According to a study of Chen et al. a widened gap between single collagen fibers in rabbit Achilles tendons frozen without surrounding medium could be observed[13]. This could not be seen in our experiments.

The evaluation of biomechanical features showed no differences between fresh tendons and tendons frozen once in PBS or DMSO indicating that the findings mentioned above are negligible by one-time freezing of tendon tissue. Similar findings have already been shown in rat Achilles tendons [20]. On the contrary, four freezing cycles in PBS led to significantly increased dynamic Young’s modulus for all strain rates compared to fresh tendons. A possible explanation might be, that interactions between tendon-residential protein such as proteoglycans and their connections via glycosaminoglycan side-chains get strengthened during the freezing process. Goh et al.[21] showed increasing stiffness in murine tail-fibers frozen at -80°C compared to unfrozen ones due to greater GAG-GAG interaction, resulting in impaired sliding of the fibrils past each other. Lower freezing temperatures (-20°C), as also used in our study, had no effect on single tail fibers. Since we detected changes in the whole tendon tissue during biomechanical testing, there might be other factors that have to be taken into consideration regarding the interactions between collagen fibers.

Tendons frozen in DMSO showed no significant increase of the measured biomechanical values. Inter-individual differences as well as the diminutive size of the sample leads to variations in the biomechanical testing routine and a relatively high standard deviation. This finding is aggravated when applying several freeze-thaw-cycles. These results resemble a work of Ng et al.[22] which showed a significant increase of tensile properties and strength of tendon tissue frozen in saline solution (o.9 per cent NaCl) at -40°C as well as porosity growth in tendons stored for 233 days in frozen state. A possible explanation was postulated to be found in strengthening and the post mortem deterioration of the collagen fibers.

It remains to be seen, whether DMSO as a cryoprotectant applied in measurements of other tendons, like the murine patellar tendon which provided most reliable and reproducible values in biomechanical testing of a tendon injury model[23], will show significant differences in our testing set-up. In addition, tendons of other species with greater thickness as murine tendons will have other rates of diffusion of DMSO through the tissue is strongly different from the diffusion through a murine Achilles tendon showing a thickness of about 0,5 to 1 mm depending on age and sex of the mice.

A review by Lansdown et al.[11] has already shown that multiple variables have an influence on tendon tissue during cryopreservation. This results in diverging postulations about the alteration of biomechanical features of tendon tissue after cryopreservation in various publications collected in the review mentioned above.

Even though there could be no differences seen in in the biomechanical features in our set-up, it highlights the question, whether gene alterations or other procedures effecting cells and their contacts to the ECM or other cells could lead to significant differences in the biomechanical analysis. Without use of a cryoprotective freezing medium these effects might vanish completely. Also, when evaluating new treatment approaches in tendon healing, preserving the cells during cryoconservation might help to understand how regenerative tissue and involved cells react to stress and rupture. Using DMSO should therefore improve experimental studies focusing on tendon repair in the clinics by providing new possibilities to analyze tendon tissue including residential and migrated cells after rupture and treatment.

The preserved integrity of the cells may be a minor factor for the overall stability but came into focus with the use of more sensitive measuring devices. A study of Hammer et al.[24] stated that decelluarized human iliotibial tracts show indifferent biomechanical features in comparison to untreated parts of the iliotibial tract, implicating that cells only play a minor role for tensile mechanics of ligaments and tendons. The study also admits that cells located between collagen fibril do contribute to load transfer by imposing constraints on lateral contraction. Consequently, cell survival could be of major importance when investigating the effects of cell-adhesion and cell-cell contact proteins in the context of tendon pathologies or examine the biomechanical properties in the enthesis a region in the tendon, which has comparably high quantities of cells and is prone to biomechanical failure.

## Conclusion

Cryopreservation with DMSO allows the biomechanical analysis of a matrix-cell-compound, since especially in areas rich of cells, such as the enthesis, cell-cell- and cell-matrix interactions could possibly influence the biomechanics, especially when gene alterations or tendon healing models are included in the biomechanical testing. A cryoprotective effect of DMSO on the tendons tissue, which is reflected in the preserved cell viability and the retained intact collagen structure shown by electron microscopy, could be shown. The use of DMSO as a medium during freezing could provide new insight into the impact of stress on cells and ECM in the tendon tissue after regeneration or tendon tissue effected by gene alterations. In summary, our results support the use of DMSO as a cryoproctectant medium during the deep-frozen storage as an alternative for the commonly used PBS, as it provides the possibility of a more physiological biomechanical testing of tendon tissue.

## Supporting information

S1 Fig Dynamic biomechanical testing showed significant differences between tendons frozen in PBS four times and freshly tested tendons for the dynamic Young's modulus. Biomechanical testing was performed using a custom-made axial testing set-up (LM1, ElectroForce/TA Instruments). Dynamic Young's modulus calculated from the amplitudes of the dynamic testing at a frequency of 0.01, 0.1,1 and 5 Hz for all strain rates
(TIF)
